# *Campylobacter jejuni* Across the Food Chain: Integrating Pathogenesis, Persistence, Antimicrobial Resistance, and Emerging One Health Control Strategies

**DOI:** 10.1155/ijm/2763026

**Published:** 2026-10-13

**Authors:** Othman Abdulrahman Mohammed, Mohammed Ali Hama, Saman A. Mohammed, Musa Muatasam Zorab, Suhaib H. Kakamad, Hazha Hassan Tahir, Amirreza Gholami, Azad Mohammed Aziz, Hamza Kamaran Hama, Kawther Hoshear Faiq, Ammar Alfergah

**Affiliations:** ^1^ Department of Medical Laboratory Science Halabja Technical College Sulaimani Polytechnic University Sulaymaniyah Iraq spu.edu.iq; ^2^ Department of Clinical Biochemistry University of Halabja Halabja Kurdistan Region Iraq; ^3^ Department of Medical Laboratory Technique Halabja Technical Institute Sulaimani Polytechnic University Sulaimani Kurdistan Iraq spu.edu.iq; ^4^ Department of Medical Laboratory Technology Bright Technical and Vocational Institute Sulaymaniyah Kurdistan Region Iraq; ^5^ Department of Medical Laboratory Technology Faculty of Health Sciences Qaiwan International University Sulaymaniyah Kurdistan Region Iraq uniq.edu.iq; ^6^ Department of Medical Laboratory Technology Azmar Technical and Vocational Institute Sulaymaniyah Kurdistan Iraq; ^7^ Department of Medical Microbiology College of Health Sciences Cihan University Sulaimaniya Sulaymaniyah Kurdistan Region Iraq sulicihan.edu.krd; ^8^ Department of Chemistry Dalhousie University Halifax Nova Scotia Canada dal.ca; ^9^ Department of Medical Laboratory Darbandikhan Technical Institute Sulaimani Polytechnic University Sulaymaniyah Iraq spu.edu.iq; ^10^ Department of Special Education Sharazur Educational College University of Halabja Halabja Iraq; ^11^ Department of Food Science and Nutrition Faculty of Agriculture and Food Ibb University Ibb City Yemen ibb-univ.net

**Keywords:** antimicrobial resistance, biofilm, *Campylobacter jejuni*, food chain, genomic surveillance, intervention, One Health, pathogenesis, poultry, viable but nonculturable state

## Abstract

*Campylobacter jejuni* continues to be a globally important foodborne pathogen and is one of the most common bacterial causes of gastroenteritis in humans. This fastidious microaerophilic organism is seldom cultured outside of animal hosts, yet it repeatedly populates poultry, livestock, water, processing environments, retail foods, domestic kitchens, and humans. Here, we critically integrate the evidence in a transmission‐connected, farm‐to‐fork pathway spanning colonization of animal reservoirs and carcass contamination to environmental persistence, human exposure, and disease (including antimicrobial resistance), detection, and intervention. In many systems, asymptomatic, high‐density colonization of poultry is the main amplification reservoir and carcass cross‐contamination, mixed‐species biofilms, aerotolerance, oxidative‐stress defense, cold survival, and viable‐but‐nonculturable cells support persistence through processing and distribution. Coordinated motility, chemotaxis, adhesion, disruption of epithelial barrier functions, invasion, and cytolethal distending toxin activity contribute to epithelial injury and inflammation, whereas lipooligosaccharide‐mediated molecular mimicry contributes to postinfectious complications. Factors such as persistent target‐site mutations, CmeABC efflux, acquired determinants, and antimicrobial selection within One Health compartments continue to sustain resistance to fluoroquinolones, tetracyclines, and macrolides in certain settings. Because no single point intervention can sufficiently control transmission across the food chain, effective risk reduction relies on a portfolio of components such as whole‐farm biosecurity, biological control, hygienic processing and handling, cold‐chain management, consumer hygiene interventions at home, antimicrobial stewardship, viability‐sensitive detection and genomic surveillance. These findings are consistent with multihurdle control strategies and integrated One Health surveillance linking both animal, food, environmental, and human health sectors. Consequently, future policy and research initiatives should ideally focus on harmonized sampling and surveillance protocols, standardized outcomes of intervention studies, validation at the commercial scale and evidence directly relating *C. jejuni* reductions to risk of human disease.

## 1. Introduction

*Campylobacter jejuni* (*C. jejuni*) occupies an odd status among foodborne pathogens. It is a nutritionally fastidious organism that grows optimally under microaerobic conditions and is more sensitive to oxygen, desiccation, and various environmental stresses [1]. However, it can be effectively transmitted via contemporary food systems and continues to be frequently linked with isolated gastroenteritis and cases of foodborne outbreaks [2]. Such an apparent discrepancy cannot be attributed to a single virulence determinant or route of person‐to‐person transmission. Instead, it mirrors the intersection of reservoir ecology, high‐level asymptomatic colonization of food‐producing animals, cross‐contamination at slaughter and processing plants, survival responses to stressors, genetic and phenotypic diversity, and ample chances for human exposure.

Poultry is at the center of this epidemiology. Intestinal colonization at high levels can occur without manifesting overt disease, enabling infected flocks to enter slaughter and processing systems without evident clinical indicators [3]. Failures during evisceration, leakage of intestinal contents, contaminated equipment, carcass‐to‐carcass transfer, and handling after carcass dressing can turn silent intestinal carriage into extensive surface contamination [4]. The risk then carries forward through retail storage and domestic handling, especially when hands, utensils, cutting boards, or ready‐to‐eat foods have been contaminated with raw poultry [5]. Ruminants, raw milk, untreated water, wildlife, companion animals, and contaminated farm environments provide additional routes linking agricultural and environmental compartments [6].

The public health implications of *C. jejuni* extend beyond the occurrence of acute diarrhea. Most infections are self‐limiting; however, complications such as severe enteritis and bacteremia in at‐risk groups, along with postinfectious sequelae (Guillain–Barré syndrome, reactive arthritis, and postinfective irritable bowel syndrome), impose additional long‐term clinical and economic burdens [7]. These findings are relevant in the context of treatment complicating factors, namely fluoroquinolone and tetracycline resistance, and emerging macrolide resistance in certain regions [8]. Antimicrobial resistance arises from multiple factors, including antimicrobial use, coselection, bacterial adaptation, and horizontal gene transfer, whereas resistant populations circulate among animals, foods, humans, and the environment [9].

Significant progress has been made in characterizing individual facets of *C. jejuni* biology. Motility, the identification of adhesins mediating attachment to host cells, mechanisms of epithelial invasion, cytolethal distending toxin (CDT) production and other virulence factors involved in pathogenesis such as lipooligosaccharide variation, oxidative‐stress defenses and biofilm‐associated survival have been investigated extensively [10]. These processes are seldom discussed collectively. The separate treatment of these stages may hide a key practical insight: The likelihood that humans become infected with *C. jejuni* depends on interactions among colonization, survival, contamination, detection, and control throughout the production continuum.

Contrary to previous reviews, which have mainly focused on or compartmentalized *C. jejuni* virulence, antimicrobial resistance, poultry interventions, environmental persistence, or One Health surveillance as independent topics, this review approaches these components in a farm‐to‐fork framework. In particular, it tracks the continuum: from animal‐reservoir colonization and on‐farm ecology, across slaughter‐ and processing‐related contamination and survival under food‐chain/environmental stresses, through retail/domestic exposure to human infection/disease. Molecular pathogenesis, antimicrobial resistance, detection, genomic surveillance, and intervention strategies are considered with regard to the points they influence on transmission and risk along the food chain. Therefore, the primary contribution of this review lies not in explaining individual mechanisms in isolation, but in integrating biological mechanisms with epidemiological evidence and surveillance approaches. Particular emphasis is focused on whether mechanistic and laboratory findings translate into measurable reductions in contamination, exposure, and human health risk. Accordingly, this review follows *C. jejuni* from animal reservoirs through slaughter, processing, retail storage, domestic handling, and human disease. Antimicrobial resistance, detection, genomic surveillance, and interventions are evaluated according to their positions within this transmission pathway.

## 2. Review Methodology

This study was carried out as a structured critical narrative review of the evidence base on *C. jejuni* within the farm‐to‐fork and One Health continuum. Literature searches were performed in PubMed, Scopus, and Embase and supplemented by searches in Google Scholar, citation tracking, and relevant reports from WHO, FAO, EFSA, and national surveillance agencies. Search terms for *C. jejuni* and campylobacteriosis were combined with terms related to reservoirs and transmission, poultry production and processing, pathogenesis, environmental persistence, stress adaptation, antimicrobial resistance, detection and genomic surveillance, interventions, and One Health. Recent literature was emphasized, although earlier seminal studies were retained when needed to provide mechanistic or epidemiological context.

Records were screened for relevance to the prespecified review domains, with potentially eligible articles assessed in full text. Epidemiological, mechanistic, surveillance, genomic, diagnostic, and intervention studies relevant to *C. jejuni* were included. Publications were excluded if they were outside the review scope or lacked sufficient relevant information or substantially duplicated findings reported more completely elsewhere.

## 3. Epidemiology and Farm‐to‐Fork Transmission Ecology

Human campylobacteriosis arises from interconnected animal, food, environmental, and domestic transmission pathways. Source‐attribution studies using epidemiological investigations, microbial subtyping, MLST, comparative genomics, and population genetic approaches have consistently identified poultry as a major source of *C. jejuni* infection in many settings [11]. However, attribution varies geographically and depends on the completeness of sampling across competing reservoirs and exposure routes [11]. Ruminants, wildlife, raw milk, untreated water, and contaminated environments may also contribute directly or indirectly to human exposure [12]. Current evidence is geographically imbalanced, with intensive poultry systems and well‐resourced surveillance networks substantially better represented than smallholder production, informal markets, conflict‐affected regions, and low‐resource settings [13]. Consequently, locally important transmission routes may be underestimated. A farm‐to‐fork perspective is therefore required to follow *C. jejuni* from animal reservoirs and farm environments through slaughter, processing, retail storage, domestic handling, and ultimately human exposure.

### 3.1. Animal Reservoirs and On‐Farm Colonization

*C. jejuni* colonizes a diverse array of avian and mammalian hosts; however, poultry represents the most direct source of routine foodborne exposure. In broilers, the organism is usually present in the ceca without clinical signs, and once introduced into a flock of susceptible birds, it may spread rapidly [14]. Colonization does not always occur immediately after placement; colonization may remain undetectable during the early growing period before becoming detectable later in production. In the presence of high stocking density, shared litter and drinking systems, insects, contaminated equipment, personnel movement, and/or partial biosecurity failures, the organism can disseminate rapidly throughout the flock once established [15, 16].

Flock colonization is multifactorial. Contaminated water, litter, insects, farm workers and equipment, neighboring livestock, wild birds, and feed are possible routes of introduction [17]. Although vertical transmission is not always as consistent as horizontal acquisition, the detection of the organism in a breeder or hatchery environment does not imply efficient vertical transfer [18]. Consequently, farm‐specific longitudinal studies are more informative than single cross‐sectional studies when identifying introduction pathways.

Genetically diverse populations excreted in the feces of cattle and sheep can contaminate pastures, soil, runoff, water, and raw milk. Other hosts, including pigs, companion animals, and wildlife, also act as reservoirs for *C. jejuni*, although the proportion contributing to human infection varies depending on location and host exposure patterns [19]. However, given the capacity of wild birds to spread organisms across broad landscapes and reintroduce them into farms, they cannot be assumed to function as direct foodborne reservoirs equivalent to poultry [20].

### 3.2. Slaughter and Processing

Slaughter represents a critical transition point at which intestinal carriage of *C. jejuni* can lead to carcass contamination. Fecal contamination before processing due to transport stress and flock mixing may increase fecal shedding and the potential for contamination. Intestinal leakage during defeathering and evisceration contaminates the skin, equipment, and water, which increases fecal contamination of carcass surfaces [21]. Reducing *C. jejuni* prevalence and intestinal loads in broiler flocks can reduce bacterial loads on carcasses at slaughter, thereby lowering the risk of human campylobacteriosis [22].

The level of contamination at the end point reflects the overall performance of scalding, defeathering, evisceration, washing, chilling, equipment hygiene, process‐water management, and line speed used, as well as flock segregation [23]. Although combined interventions generate substantial reductions, the remaining population may still be relevant because of a generally low infectious dose and secondary transfer opportunities in the downstream handling process [21]. Process control should therefore emphasize cumulative reduction and prevention of recontamination rather than reliance on a single lethal step.

The presence of organic residues or established microbial communities on food‐contact surfaces can impair the effectiveness of sanitation routines, resulting in recurrent contamination [24]. However, the detection of *C. jejuni* on equipment should be interpreted with caution, as persistence may reflect recurrent introduction from contaminated carcasses, protection within mixed‐species communities, or survival in organic residues rather than the establishment of a stable monospecies biofilm under aerobic processing conditions [25].

### 3.3. Milk, Water, Wildlife, and Environmental Circulation

Contaminated raw milk can result from direct fecal contamination, dirty udders or udder anatomy, insufficient cleaning of equipment, or improper storage [26]. Because *C. jejuni* is unlikely to multiply in properly refrigerated milk, the risk is determined by the initial contamination level, survival, unpasteurized consumption, and delay between contamination and ingestion [26]. If done correctly, pasteurization remains a very effective control [27].

Water serves as a conduit for exposure and also a connector in ecosystems. *C. jejuni* can access surface and private water systems through fecal inputs from wildlife, livestock, or source water, including wastewater and agricultural runoff [11]. Environmental survival is influenced by temperature, light, and oxygen availability [28]. Rainfall and flooding cause the mobilization of fecal contamination, which links farms, water sources, wildlife habitats, and human communities [27, 29].

Environmental detection does not directly transmit to humans; however, it can reveal circulation pathways and identify points where interventions in one sector intersect with those in another. Consequently, One Health surveillance should consider environmental sampling as an adjunct to source attribution rather than a standalone descriptive exercise.

### 3.4. Retail, Storage, and Domestic Handling

Factors influencing the prevalence and concentration of *C. jejuni* in retail poultry products include upstream flock colonization, processing hygiene, product type and postprocessing packaging and storage conditions. In contrast to *Listeria monocytogenes*, *C. jejuni* cannot grow at normal refrigeration temperatures but can survive in the refrigerated state for extended periods of time allowing for transmission [30, 31]. Therefore, refrigeration primarily restricts bacterial multiplication rather than eliminating contamination. Freezing has a potential effect on characteristics of *C. jejuni* populations in poultry meat, as it can decrease the number of viable bacteria recovered following thawing; however, freezing may not confidently eliminate this organism and should not be considered a decontamination step [32]. The survival of *C. jejuni* in poultry products may also be dependent on packaging atmosphere, storage temperature and oxygen availability as a secondary factor beyond sufficient hygienic processing and proper temperature control [33].

The domestic kitchen is an important last point of exposure for consumers to *C. jejuni*, especially at the time of handling and preparation of raw poultry products [34–36]. Although sufficient cooking inactivates *C. jejuni* present in poultry meat, it may result from cross‐contamination prior to cooking of bacteria from raw poultry to hands, utensils, food‐contact surfaces or ready‐to‐eat foods [35]. As risk reduction to consumers depends on practical practices, that is, separating raw poultry from ready‐to‐eat foods, washing hands [35], cleaning utensils and food‐contact surfaces, preventing cross‐contamination, cooking thoroughly, and avoiding unpasteurized milk [36]. If consumers are to be advised, this advice should priorities the most specific time‐ and culturally‐appropriate hygiene behaviors over general tips to simply “cook properly.” Table 1 outlines the primary reservoirs, vehicles and transmission points of human infection with *C. jejuni* along with information pertaining to source‐attribution evidence, public health relevance, and important uncertainties (Figure 1).

**Table 1:** Principal sources, food and environmental vehicles, and transmission nodes for *C. jejuni* illness, showing major exposure routes, supporting source‐attribution evidence, major public health relevance, and key uncertainties.

Reservoir, vehicle, or transmission node	Principal pathway	Representative evidence	Public‐health significance	Interpretive limitation/key uncertainty	References
Animal reservoirs					
Broiler poultry and poultry meat	Asymptomatic cecal colonization followed by contamination during slaughter, retail handling, and kitchen cross‐transfer is common.	Case‐control, outbreak, MLST/WGS, and attribution studies consistently identify poultry as the leading source of many outbreaks in industrialized settings.	Principal amplification reservoir and routine food‐chain source	Attributable fractions vary with geography, source sampling, production, processing, diet, and the analytical method.	[11, 12, 21, 37, 38].
Cattle and sheep	Fecal shedding, direct contact, runoff, raw milk contamination, and consumption of unpasteurized milk.	Outbreak, epidemiological, and genomic evidence support ruminant‐associated infection through raw milk, water, and direct contact.	Important secondary reservoirs, locally prominent where livestock exposure or raw milk consumption is common.	Contributions may be underestimated when livestock, milk, water, and human isolates are not sampled contemporaneously.	[11, 12, 19, 38].
Wild birds and other wildlife	Fecal contamination of water, pasture, feed, litter, soil, and farm environments.	Ecological and genomic studies demonstrate carriage and dissemination, but direct attribution to human disease is less consistent than that to poultry.	Ecological connectors among wildlife, farms, livestock, and water systems.	The transmission direction is often unresolved and may be bidirectional.	[12, 19, 20, 28].
Companion animals and direct contact	Contact with pets, puppies, livestock, feces, or contaminated animal environments.	Case‐control, outbreak, and genomic evidence support transmission in specific populations and resistant lineages.	This factor, often regarded as secondary, is significant in domestic settings, veterinary contexts, agricultural environments, and in outbreaks associated with specific lineages.	Direct transmission may be difficult to distinguish from shared environmental or common source exposure.	[19, 38–40].
Food and environmental vehicles					
Raw milk and dairy environments	Fecal or udder contamination, inadequate equipment sanitation, poor storage, and consumption without pasteurization.	Recurrent epidemiological and outbreak evidence has identified raw milk as an episodic but established vehicle.	Infrequent contamination can lead to substantial outbreaks when exposure is widespread.	The relative contributions of shedding, milking hygiene, equipment, storage, and handling are difficult to separate.	[26, 27, 38].
Water and wastewater	Contamination of wells, surface water, recreational water, and drinking water systems can occur due to livestock, wildlife, wastewater, or runoff.	Environmental surveillance, outbreak investigations, source tracking, and genomic comparisons collectively indicate the transmission and circulation of pathogens via waterborne routes.	Direct exposure to vehicles and bridges among animal, agricultural, environmental, and human compartments.	DNA detection does not establish the viability, infective dose, or direct human transmission.	[11, 12, 28, 29].
Transmission and exposure nodes					
Slaughter and processing	Defeathering, evisceration leakage, contaminated equipment or water, and carcass‐to‐carcass transfer.	Processing studies consistently demonstrate the redistribution of contamination from colonized flocks.	Critical transition converting intestinal carriage into contaminated products.	Recurrent contamination may reflect repeated introduction, environmental survival, biofilm formation, or a combination of these factors.	[21, 25].
Retail storage and domestic handling	Survival during storage, inadequate cooking, package leakage, and transfer to hands, utensils, surfaces, or ready‐to‐eat food.	Evidence obtained from retail surveys, risk assessments, and kitchen studies indicates the presence of both direct ingestion and indirect cross‐contamination.	Final exposure interface: Cross‐contamination may occur even if the poultry is subsequently cooked adequately.	The risk varies with the starting load, storage, transfer efficiency, cooking, behavior, and hygiene.	[5, 32, 36].

**Figure 1:**
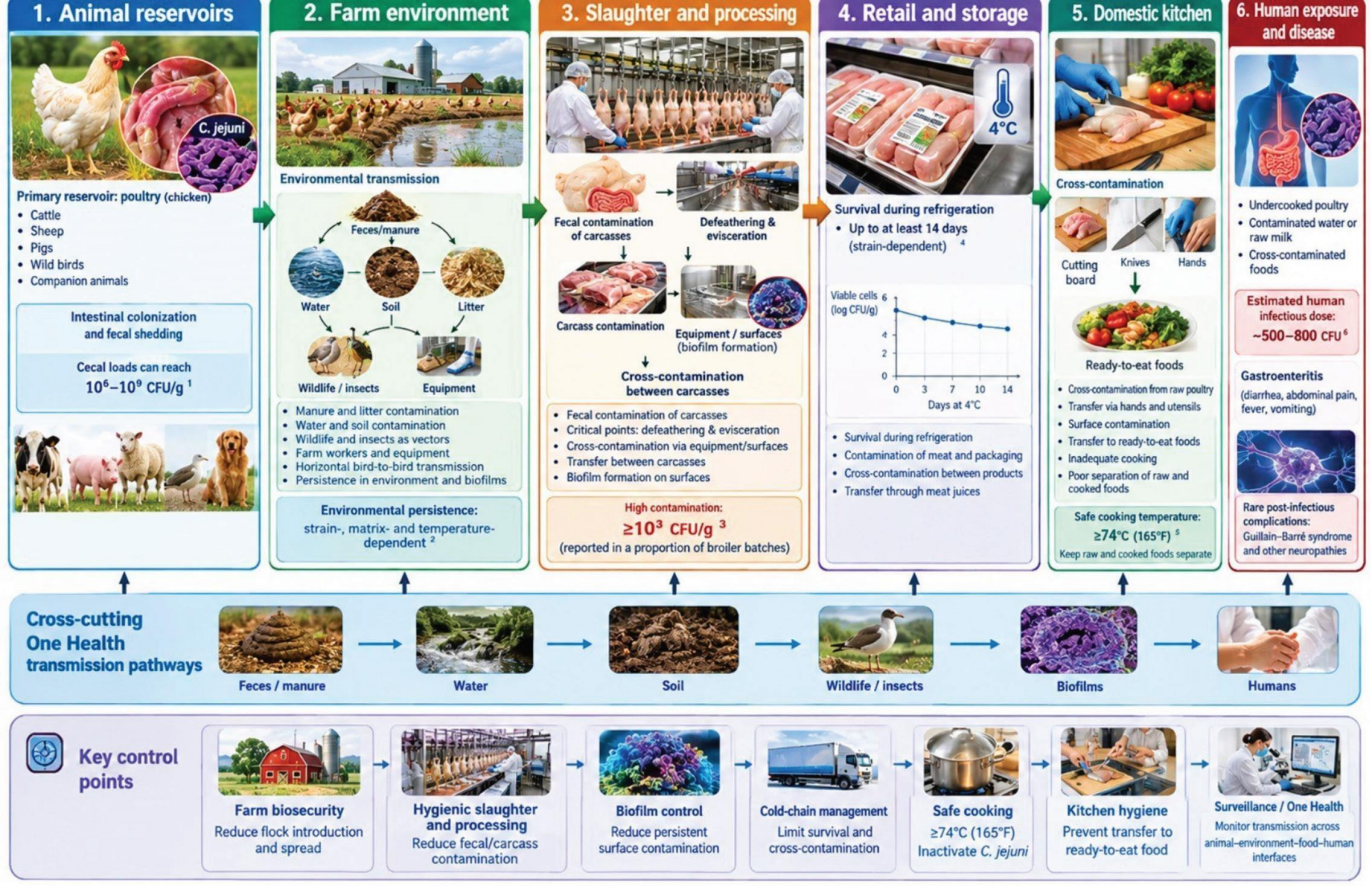
Farm‐to‐fork transmission ecology of *Campylobacter jejuni*. It illustrates the transmission pathway of *C. jejuni*, which can occur from animal and environmental reservoirs to humans through several stages: on‐farm colonization, slaughter and processing, retail storage, and domestic food handling.

## 4. Molecular Basis of Colonization and Human Disease

The pathogenesis of human campylobacteriosis involves the concerted action of several bacterial factors responsible for intestinal navigation, adherence to epithelial surfaces, breach of epithelial barriers, and subsequent injury that may be mediated either through direct invasion or toxin production followed by host immune modulation. This chapter thus considers how these mechanisms work both individually and together to initiate colonization, cause acute intestinal disease, and contribute to postinfectious complications.

### 4.1. Motility, Chemotaxis, and Mucus Navigation

The intestinal environment presents an obstacle that must be successfully navigated to establish pathogenesis. The bipolar flagella facilitate motility in a thick mucus environment, and chemotaxis systems direct movement towards nutrients and host‐derived signals [41]. Motility assistances reach to intestinal crypts, enhance contact with epithelial surfaces, and participate in successful intestinal colonization. Flagellar glycosylation, phase variation, and regulation further modify performance to adapt to changing host conditions [42].

The flagellar export apparatus also participates in the secretion of invasion‐associated Cia proteins. Experimental evidence indicates that these proteins are secreted through the flagellar export. Therefore, this is more accurately described as a flagellum‐associated secretion function than as a conventional, independently encoded Type III secretion system [43]. This distinction is important because it links motility and host cell interaction while avoiding mechanistic overstatement [10, 44].

### 4.2. Adhesion and Epithelial‐Barrier Disruption

After mucus penetration, adhesion stabilizes the bacterial contact with epithelial cells and extracellular matrix components. CadF and FlpA bind fibronectin and contribute to the downstream signaling required for efficient invasion [45]. Other surface proteins, including JlpA, PEB1, CapA, and strain‐dependent adhesins, may contribute to host and tissue specificity [46]. The relative importance of individual determinants depends on the strain, host model, and assay used; the relative contribution of individual adhesins may be limited by functional overlaps between surface structures [47].

Barrier disruption can occur through serine protease HtrA and related host–pathogen interactions, which affect junctional proteins. This facilitates paracellular transmigration and exposes the basolateral receptors and fibronectin, which are less accessible from the luminal surface [42]. Therefore, epithelial damage is produced by coordinated bacterial contact, proteolysis, host signaling, and inflammation, rather than by a single toxin [48].

### 4.3. Invasion and Intracellular Trafficking

Adhesion activates host signaling pathways that remodel the actin cytoskeleton and promote bacterial uptake [48, 49]. *Campylobacter* invasion antigens exported through the flagellar apparatus contribute to membrane ruffling and internalization [42, 44]. Specific pathways differ across epithelial models, and it is inappropriate to generalize findings from immortalized cell lines to the intact human intestine.

Internalized bacteria can inhabit structures known as “Campylobacter‐containing vacuoles,” where they may temporarily evade effective degradation by the lysosomal apparatus. Persistence within these niches is short‐lived and strain‐specific but contributes to prolonged contact with softer tissues, intensifying the signaling of inflammation [42, 47]. There is little evidence for the extreme view that intracellular localization makes the organism immunologically invisible; invaded epithelial cells and immune cells respond with vigorous inflammatory responses.

### 4.4. CDT and Inflammatory Injury

The CDT is a tripartite genotoxin composed of CdtA, CdtB, and CdtC [50]. The Cdt holotoxin consists of three peptides: The polypeptides CdtA and CdtC are involved with binding and delivery, whereas CdtB is the DNase‐like enzymatically active component. The nuclear entry of CdtB induces DNA damage, the activation of checkpoints and either cell cycle arrest or death/senescence‐like responses [51]. Intestinal dysfunction in CD is influenced by CDT but also importantly impacted by the level of bacterial invasion, relative susceptibility of the host and extent of the inflammatory response [52].

Recognition of bacterial surface structures and intracellular signals modulates cytokine production, neutrophil recruitment to the site of invasion, barrier injury, and symptoms of diarrhea. While promoting bacterial clearance, exaggerated inflammatory responses may aggravate tissue injury [53]. The clinical phenotype arises from the interaction between bacterial virulence and epithelial responses, innate immune activation, and host regulatory responses [52, 54]. The terminology and evidence remain context‐dependent; therefore, claims regarding pyroptosis or specific cell death pathways should be linked to a precise experimental model.

### 4.5. Surface Variation, Molecular Mimicry, and Postinfectious Autoimmunity

Antigenic diversity between and within strains is generated through phase‐variable expression and modification of lipooligosaccharides, capsular polysaccharides, flagellar structures, and outer‐membrane proteins [55]. These differences have the potential to change complement sensitivity, specificity for antibody binding and colonization and tissue interaction. Therefore, capsular and LOS structures should not be merely labeled as stealth factors; instead, they are modifiers of host recognition that can simultaneously stimulate powerful inflammatory responses [54].

Some strains contain sialylated LOS structures that mimic human peripheral‐nerve gangliosides [47]. Infection can lead to the production of cross‐reactive antiganglioside antibodies, complement activation, and nerve injury, with mechanistic ties to Guillain–Barré syndrome and related neuropathies [56]. Molecular mimicry thus represents an autoimmune consequence of immune recognition, not just a strategy to escape detection. Host genetics, the infecting LOS structure, and immune regulation influence the probability and clinical form of the complication. The sequential roles of the major pathogenic determinants during human *C. jejuni* infection are integrated in Figure 2.

**Figure 2:**
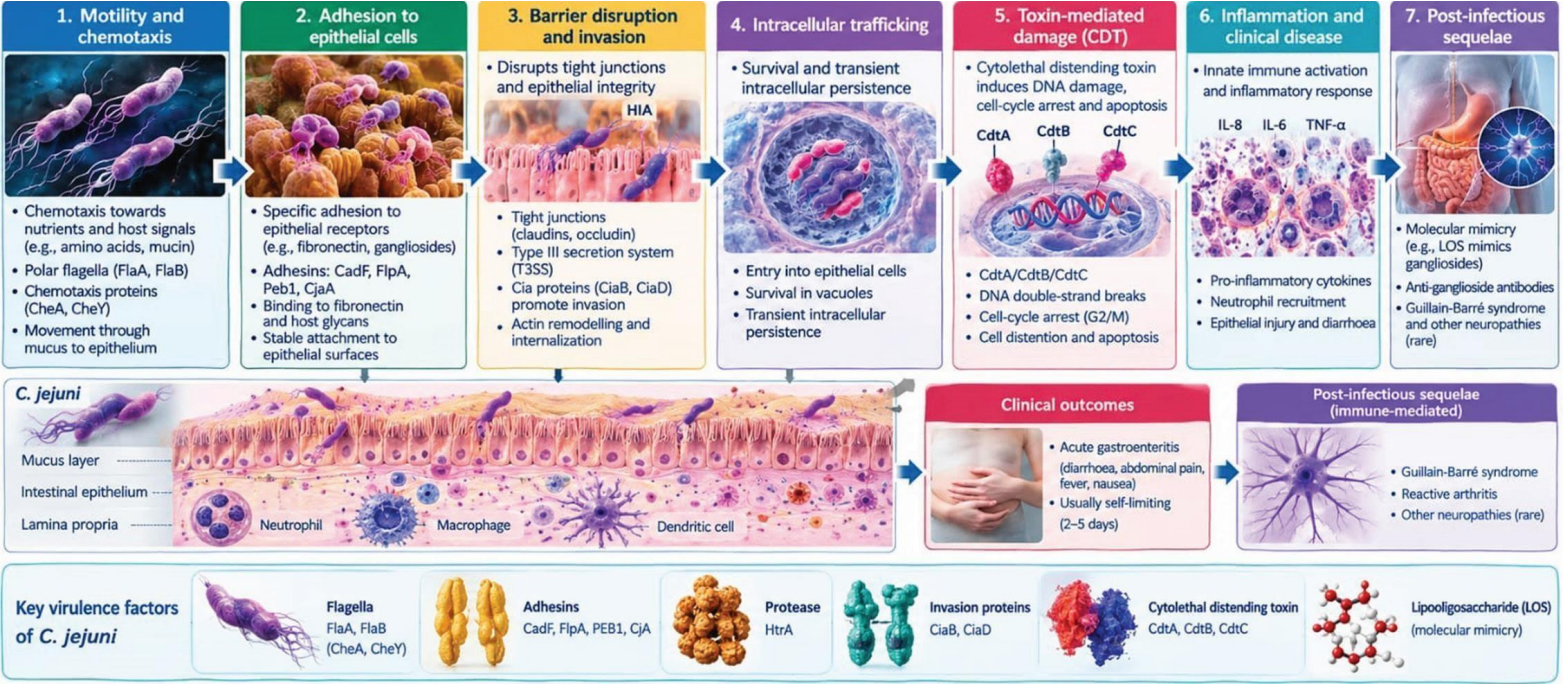
Molecular pathogenesis of *C. jejuni* in humans. This figure summarizes the sequential roles of motility and chemotaxis, epithelial adhesion, barrier disruption and invasion, transient intracellular trafficking, CDT‐mediated DNA damage, inflammatory injury, and immune modulation, leading to postinfectious complications.

## 5. Environmental Persistence and Food‐Chain Stress Adaptation

Although *C. jejuni* does not usually multiply extensively outside its host, it can remain viable throughout food production and distribution by adapting to oxygen exposure, refrigeration, nutrient limitation, acidic conditions, and other environmental stresses. This section investigates the mechanisms that support persistence in external environments.

### 5.1. Survival Without Sustained Environmental Growth

The ecological success of *C. jejuni* is based primarily on its survival and repeated transmission, rather than robust multiplication, in most external environments [10, 30]. The organism encounters oxygen, refrigeration, heat, nutrient limitation, acidic pH, temperature fluctuations, and antimicrobials. Survival is highly strain dependent, and laboratory reference strains may not represent hyperaerotolerant or stress‐adapted lineages circulating in food systems [1, 57].

Aerotolerance is particularly important because processing and retail environments expose microaerophilic organisms to atmospheric oxygen [31]. Catalase, superoxide dismutase, alkyl hydroperoxide reductase, peroxidases, DNA repair systems, metal homeostasis, and regulatory responses collectively reduce oxidative damage [58, 59]. These systems do not make the bacterium conventionally aerobic; they extend survival during transient or localized exposure and can produce cross‐protection against other stresses [60].

### 5.2. Biofilms and Mixed‐Species Communities

Biofilm‐associated growth is most relevant to persistence on farms and processing surfaces, not as a separate stage of human pathogenesis. Initial attachment is influenced by motility, surface proteins, extracellular DNA, organic residues, surface material, temperature, oxygen, and the surrounding community. Under experimental conditions, mature biofilm structures may protect cells from oxidative stress, nutrient limitation, and disinfectants. However, the magnitude of protection varies widely among strains, environmental conditions, and experimental models [24, 47].

Mixed‐species communities may be more ecologically relevant than monospecies biofilms. Oxygen utilizing obligates or facultative microorganisms may utilize oxygen, release extracellular matrix or alter the availability of nutrients and generate protective microenvironments that enhance the survival of *C. jejuni* [61, 62]. Although associations with other processing‐environment bacteria have been detected, the functional contributions of each partner should be experimentally investigated.

### 5.3. Cold, Acid, and Other Types of Stress: Cold Stress Osmotic Stress Nutrient Deprivation

Although refrigeration inhibits multiplication, the pathogen is usually still viable long enough to be transmitted. Survival in cold is not controlled by one universal pathway but depends on establishing adaptive mechanisms, such as biofilm formation and a process referred to as the viable but nonculturable state, in response to stress exerted by freezing temperature and/or fluctuation of temperatures. Thus, cold‐chain management should be considered a growth‐control and partial reduction measure rather than minimization [31].

The survival of *C. jejuni* is influenced by environmental stressors, such as exposure to acid from foods and through gastrointestinal transit, osmotic stress, and nutrient deprivation. The degradation of metabolic pathways and conservation of energy resources confer a certain degree of survival under these adverse conditions; however, the physiological state of cells, nutrient composition of the media, pre‐existing stress response history, and strain background all influence whether prosurvival responses or programmed cell death will prevail [63, 64].

### 5.4. Viable but Nonculturable Cells

Populations can lose certain colony‐forming abilities on routine media, but at the same time maintain selected indicators of viability under prolonged oxidative, cold, osmotic, or nutrient stress. This state is referred to as viable but nonculturable condition. Coccoid cells may decrease metabolic activity, change gene expression, and maintain membrane integrity or transcripts. Nevertheless, there is no one measure that demonstrates complete physiological viability or infectivity [65, 66].

The role and implications of VBNC cells in food safety, however, cannot yet be extrapolated from membrane integrity, metabolic activity or molecular detection alone. Some suggest retention of virulence‐associated activity, whereas others indicate progressive cellular injury and loss of infectivity with strain, induction conditions, duration, resuscitation approach, and experimental model from study to study [67]. Therefore, detection of a VBNC‐like state should not itself be interpreted as evidence of infectivity. The most defensible conclusion is that culture‐based methods could miss stressed populations, whereas molecular detection of DNA only may inflate their food‐safety significance. Where possible, risk assessment should be based on a combination of culturability, membrane integrity, metabolic activity, and evidence of biological recovery or infectivity [68].

## 6. Antimicrobial Resistance: Mechanisms, Epidemiology, and One Health Surveillance

Antimicrobial resistance in *C. jejuni* may result from target‐site mutations, acquired resistance determinants, efflux activity, and antimicrobial selection in humans, animals (including livestock), food, and environmental settings. Understanding these integrated mechanisms and transmission channels is essential for interpreting regional resistance patterns and strengthening the One Health surveillance.

### 6.1. Clinical Relevance and Therapeutic Implications

Simple *C. jejuni* infections are usually self‐limiting and treated predominantly with oral or intravenous fluid replacement and electrolytes as needed. However, antimicrobial therapy may be indicated in cases with severe or persistent enteritis, bacteremia, extraintestinal infection, risk during pregnancy, immunocompromising conditions, including age in older adults, and clinically significant comorbidities [69]. When antimicrobial therapy is indicated, azithromycin is generally the first‐choice treatment for *Campylobacter* infection [70]. Fluoroquinolones may also be used in certain settings, but the empiric utility of this class has been greatly diminished by widespread ciprofloxacin resistance [71]. Thus, susceptibility testing is especially relevant in the setting of severe, recurrent, invasive, or treatment‐resistant infections.

Resistance phenotypes should be interpreted in their clinical and epidemiological context rather than considered in isolation. In *C. jejuni*, fluoroquinolone resistance is numerically dominant in many surveillance systems, whereas macrolide resistance is less common [72]. However, macrolide resistance is of specific clinical importance because it eliminates crucial therapeutic options. Tetracycline resistance is widely observed, especially in food‐animal isolates [73]. Multidrug‐ and extensively drug‐resistant lineages represent an additional challenge, as combinations of target‐site mutations, acquired determinants, and heightened efflux can target both first‐line and alternative agents [40].

### 6.2. Regional and Source‐Specific Resistance Patterns

Resistance patterns in *C. jejuni* are extremely heterogeneous by region, host, source, antimicrobial class, and surveillance system. Ciprofloxacin and tetracycline resistance remain high in humans, particularly among isolates in many reporting countries, as shown by European One Health surveillance reports from recent years [71, 74]. Macrolide resistance is generally lower but remains clinically important because macrolides are key therapeutic agents [75]. Tetracycline resistance frequently accompanies fluoroquinolone resistance, particularly among food‐animal and poultry‐associated isolates [75, 76].

The collection of human, retail meat, and food animal isolates in the United States through NARMS is similar, but direct comparisons with published European estimates have been difficult because of substantial differences in sampling, testing, and interpretive techniques [77, 78]. Other features that highlight how aggregate surveillance data can hide epidemiologically important lineages are the aforementioned resistant subpopulations, including the highly drug‐resistant REPDBR01 lineage, primarily associated with puppy exposure [39]. Nomenclature for estimates of regional resistance should therefore focus on the bacterial species and isolate source, surveillance design, testing method, and year of reporting rather than being compared as directly equivalent prevalence values.

### 6.3. Target‐Site Modifications and Other Resistance Determinants

The fluoroquinolone resistance of *C. jejuni* is mainly associated with gyrA mutations, especially Thr‐86‐Ile, which weakens the interaction between ciprofloxacin and DNA gyrase. *C. jejuni,* like many gram‐negative bacteria, does not have the classical topoisomerase IV; therefore, resistance is less frequently attributed to *parC* mutations [79]. Resistance to macrolides is primarily mediated by mutations in the 23S rRNA, especially A2074C/G and A2075G; however, it can also be due to L4/L22 alterations or the presence of a transferable erm(B) determinant [73, 80]. Importantly, the detection of a resistance determinant does not correlate with the phenotype in an absolute manner, as expression and genomic context can alter susceptibility [81]. Tet(O) and related ribosomal protection determinants are the most common mediators of tetracycline resistance [82]. In contrast, OXA‐type *β*‐lactamases and antimicrobial‐modifying enzymes are responsible for resistance to *β*‐lactams and aminoglycosides [83, 84]. These mechanisms can accumulate to produce multidrug‐resistant profiles in bacteria.

### 6.4. Efflux and Genetic Exchange: Markers for Bacterial Fitness

The CmeABC efflux system mediates intrinsic and acquired decreased susceptibility by exporting many different types of antimicrobial and toxic compounds, including fluoroquinolones, macrolides, and tetracyclines [62]. CmeABC primarily functions as a resistance modifier that can act in synergy with gyrA or 23S rRNA mutations, other acquired resistance genes alone or together; further upregulation may enhance efflux activity [85, 86]. At least partly due to the effects on relative fitness, some mutations are more stable over time than others, and further genes associated with resistant genotypes can remain stably maintained for long periods in the absence of selection pressure provided by antimicrobial use [79, 87]. Consequently, antimicrobial stewardship may not alone reverse resistance when successful resistant lineages become established in animal hosts and food‐chain environments. The principal mechanisms and interpretive limitations are summarized in Table 2.

**Table 2:** Principal antimicrobial resistance mechanisms in *Campylobacter jejuni*, including their molecular targets, genetic determinants, mechanistic effects, clinical and surveillance significance, and key interpretive limitations.

Antimicrobial class or phenotype	Primary target	Major determinants	Mechanistic effect	Clinical/surveillance significance	Interpretive limitation	References
Fluoroquinolones	DNA gyrase	QRDR mutations in *gyrA*, especially Thr‐86‐Ile; increased CmeABC efflux	GyrA substitution reduces binding to DNA gyrase, and efflux lowers intracellular exposure and can amplify resistance.	The substantial resistance to ciprofloxacin observed in numerous human and food‐animal systems restricts its empirical use.	*C. jejuni* lacks conventional topoisomerase IV and does not routinely attribute resistance to *parC*. Uncommon *gyrA* variants are context‐dependent.	[79, 85].
Macrolides	50S ribosomal subunit	23S rRNA A2074C/G and A2075G; erm(B); selected L4/L22 changes; CmeABC	Target alteration or methylation reduces ribosomal binding, and efflux can increase resistance levels.	Usually less prevalent than fluoroquinolone resistance but more therapeutically consequential.	Some L4/L22 variants are lineage polymorphisms, and determinant detection alone does not establish the phenotype.	[81, 88].
Tetracyclines	30S ribosomal subunit	Tet(O) and related variants, chromosomal or mobile	Tet(O) protects the ribosomes and permits translation.	Widespread, especially in poultry and food animal isolates; often co‐occurs with fluoroquinolone resistance.	Interpretation of MIC, gene integrity, expression, and genomic context.	[73, 82].
*β*‐lactams	Penicillin‐binding proteins	*blaOXA*‐family alleles, including *blaOXA-61*; altered permeability; CmeABC	*β*‐lactamase hydrolysis and reduced drug access to periplasmic targets.	Heterogeneous susceptibility complicates therapeutic and genomic inferences.	The presence of blaOXA alone is insufficient to predict resistance, as factors such as promoter activity, gene expression, allele variation, specific agent, and minimum inhibitory concentration (MIC) are also significant.	[83]
Aminoglycosides	30S ribosomal subunit	*aph*, *aac*, *aad*, and related modifying‐enzyme genes	Phosphorylation, acetylation, and adenylation reduce drug activity.	Although less prominent overall, determinants may accumulate in mobile MDR regions.	Substrate profiles differ; confirmation with antimicrobial‐specific phenotypic testing is required.	[84, 89].
Multidrug resistance (MDR)	Multiple targets	Combinations of target mutations, acquired genes, increased efflux, plasmids, and genomic islands	Multiple mechanisms confer resistance to antimicrobial classes.	MDR/XDR lineages reduce first‐line and alternative treatment options.	The definitions differ according to the panel, criteria, affected classes, species, and source of the isolate.	[39, 40, 85, 90].

### 6.5. Selection and Dissemination Across One Health Compartments

In human medicine, veterinary practice, and food animal production, antimicrobial use applies direct selection pressure for resistant *C. jejuni*. Selection can be a population‐level phenomenon that occurs when active treatment (therapeutic treatment or metaphylaxis) and historical exposure to antimicrobial agents are present. These resistant strains, when established, can spread along food supply chains from production farms to animal transport and slaughter, processing plants, retail foods, and through direct contact with animals onto the surrounding environment [19, 91].

The link between antimicrobial usage and resistance is not confined to the direct administration route. Human and animal waste can contain residues of antimicrobials, resistant bacteria, and resistance genes from the reuse of manure, agricultural runoff, wastewater, surface water, soil, and sediment, which can act as reservoirs for protection [92, 93]. In cases where resistance determinants are genetically or physiologically linked in these systems, this may lead to the coselection of metals, disinfectants, or other pollutants [94].

Although evidence demonstrates that *C. jejuni* can be detrimental to poultry health and performance [95], poultry is particularly important because resistant populations can be amplified by high‐density colonization. These resistant isolates can cross‐contaminate carcasses during defeathering and evisceration, leading to their introduction into retail spaces and homes [90]. Resistance from human and poultry isolates clustered into similar resistance profiles, suggesting a possible epidemiological connection; however, the resistance phenotype alone cannot prove direct transmission [19, 39]. The detection of common ancestry, indirect dissemination in the environment, and direct transmission through the food chain requires high‐resolution genomic analyses, accompanied by relevant exposure data.

### 6.6. Phenotypic, Genomic, and Integrated Surveillance

This is because phenotypic antimicrobial susceptibility testing, which measures the actual response of *C. jejuni* to antimicrobial agents in a given environment, remains crucial [96–98]. Broth microdilution provides MIC values that can be used according to clinical breakpoints or epidemiological cutoff values [81]. It is plausible that novel alleles, expression differences, and genetic interactions may influence genomic prediction [98], so candidate genotypes for resistance must be constantly calibrated against standardized phenotypic antimicrobial‐susceptibility data [99]. The detection of emerging resistant lineages and the interpretation of resistance trends can benefit from integrated One Health surveillance, which encompasses human, animal, food, and environmental sources [100, 101].

Nevertheless, comparisons remain limited by differences in bacterial species, antimicrobial panels, sampling frames, MIC ranges, and breakpoint systems. All resistance estimates should then include the bacterial species, isolate source, region, surveillance year, numerator and denominator, susceptibility‐testing method and interpretive standard. Some quantitative ecological, clinical and surveillance One Health relationships are integrated here in Figure 3.

**Figure 3:**
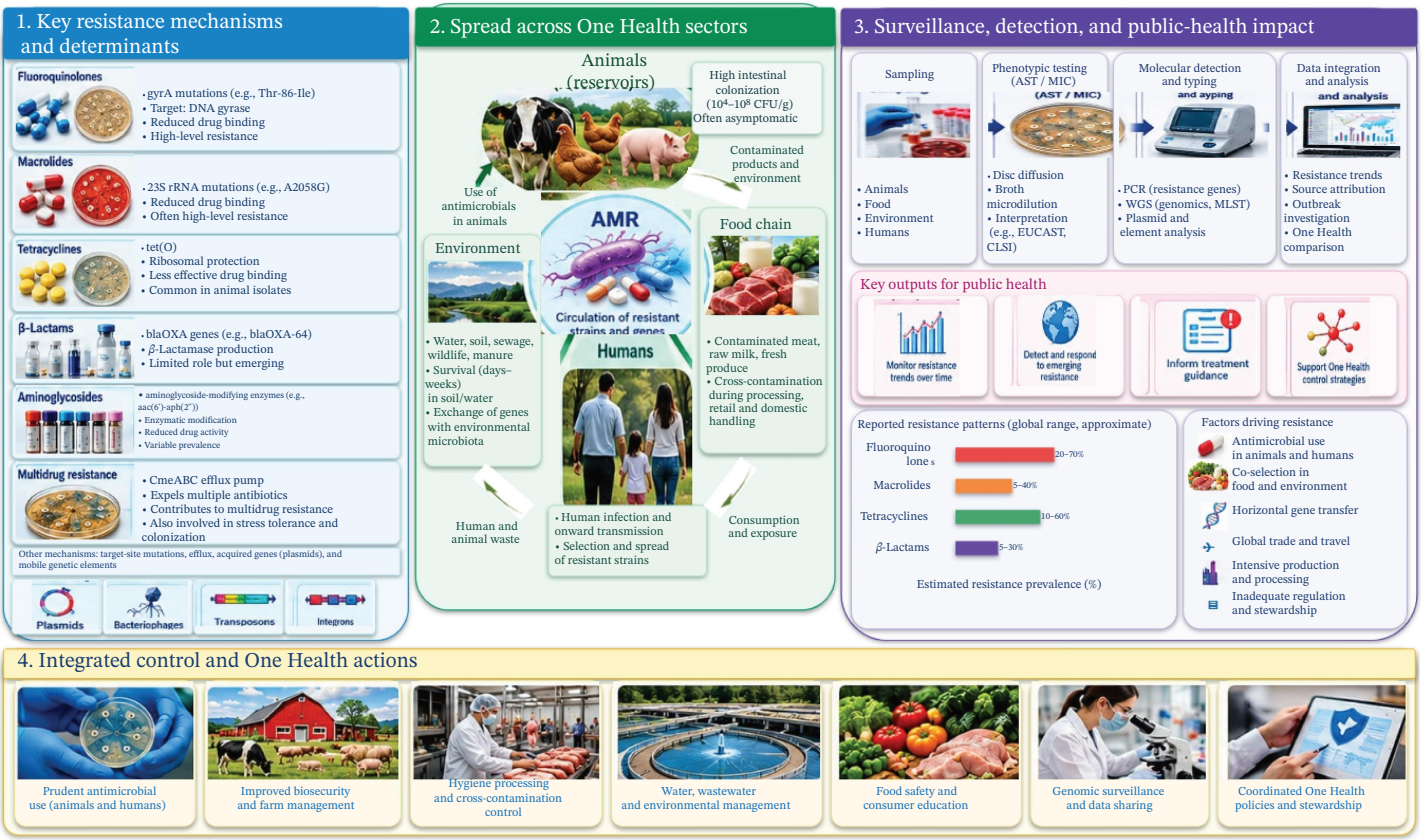
Antimicrobial resistance in *C. jejuni*. This figure summarizes the key molecular mechanisms of antimicrobial resistance, how resistant strains spread through interconnected One Health sectors (animals, food, environment, and humans), and how integrated surveillance can inform treatment decisions, monitor signals of resistance trends over time, assist in outbreak investigation and source attribution for resistant strains, and strategies for antimicrobial stewardship.

## 7. Intervention Strategies Across the Production Continuum

Effective control of *C. jejuni* necessitates coordinated interventions throughout the production continuum, as no single measure can reliably prevent colonization, contamination, persistence, and human exposure. Reported efficacy must be interpreted with respect to the experimental matrix, endpoint, treatment conditions, and validation status, since reductions in broth or controlled animal models do not automatically indicate lower carcass burden or human disease [102, 103]. Therefore, intervention strategies should be assessed not only for their microbiological effectiveness but also for their translational relevance under commercial conditions.

### 7.1. Farm Biosecurity and Flock‐Management Interventions

Farm biosecurity constitutes a critical control measure in the preharvest stage, emphasizing the prevention of pathogen introduction and dispersal [104]. Measures include restricted farm access, dedicated clothing and equipment, drinking‐water hygiene, pest and insect control, effective cleaning between flocks, litter management, separation from other livestock, and all‐in/all‐out production where feasible [105].

The principal outcome of biosecurity is usually reduced flock prevalence or delayed onset of colonization rather than a defined log_10_ reduction. This distinction is important because later acquisition can reduce the period available for within‐flock amplification before slaughter [106]. However, the reported effectiveness varies among farms because biosecurity is implemented as a behavioral and operational system rather than as a standardized dose. This effect may also be undermined by contaminated insects, equipment, water, neighboring animals, or incomplete cleaning between production cycles [105].

Commercial relevance is high because many measures are already incorporated into good agricultural practice. Nevertheless, efficacy depends heavily on adherence, farm design, labor availability, climate, production intensity, and surrounding reservoir pressure [106, 107]. Studies should therefore avoid attributing flock negativity to one measure when several biosecurity practices were introduced simultaneously.

### 7.2. Probiotics, Prebiotics, Competitive Exclusion, and Postbiotics

Microbiota‐directed interventions can reduce intestinal *C. jejuni* colonization through competitive exclusion, antimicrobial metabolite production, and modulation of intestinal barriers and immune function. The reported effects in experimental broilers are highly variable, ranging from negligible reductions to several‐log decreases in cecal populations under favorable conditions [108, 109]. Efficacy depends on the formulation, administration strategy, host microbiota, and challenge conditions, and reductions may be transient, with bacterial rebound before slaughter [110, 111]. Commercial‐scale validation remains limited, whereas formulation stability, manufacturing consistency, and regulatory requirements are important constraints [112]. Nevertheless, probiotics, prebiotics, and postbiotics should be distinguished because they differ in composition, mechanisms, and regulatory status [113, 114].

### 7.3. Bacteriophages

Bacteriophages provide strain‐specific lysis of *C. jejuni* and are particularly attractive for administration shortly before slaughter. Experimental cecal reductions are often dramatic, but, as mentioned previously, they would be transient with rebounding bacterial populations [115]. Efficacy is chiefly limited by the phage target range, dosing, timing, stability in the gastrointestinal tract, and diversity of the population being targeted [116]. Various phage cocktails could widen coverage and prevent resistance, although synergistic effects are not to be taken for granted [117]. Challenges of field‐strain diversity, resistance evolution, formulation and delivery, manufacturing quality, and regulatory matters continue to constrain commercial application [118].

### 7.4. Vaccination

We discuss how vaccination might decrease intestinal *C. jejuni* colonization at slaughter without direct antimicrobial selection pressure. Vaccines based on a range of approaches are currently available or under development, including recombinant, vectored, attenuated, outer membrane, and multivalent vaccines [119]. Published efficacy can range from low to high due to variations in antigenic diversity, host, and challenge, which are affected by the short broiler production cycle [120]. Many candidates are still in the exploratory stage, and their translation to success will depend on robust and rapid protection, effectiveness against strains that vary substantially over time and geography, and compatibility with large‐scale deployment [121].

### 7.5. Slaughter and Processing Interventions

The processing interventions typically provide more rapid and reproducible reductions than preharvest biological strategies because treatment conditions can be standardized to a greater degree. However, their effectiveness depends on the carcass contamination level, skin structure, organic load, temperature, concentration contact time, and water quality, as well as line speed [122].

Techniques such as scalding, washing, very rapid chilling, freezing, steam, and chemical decontamination can reduce viable counts when applied alone or in combination; however, complete elimination is unusual. Reductions reported are frequently higher in samples inoculated artificially than in naturally contaminated carcasses [123]. High inactivation can be provided via thermal or steam treatment; however, overtreatment may lead to different skin appearances, textures, moisture retention, and potentially yields [124].

When used under appropriate conditions (i.e., concentration and contact time), organic acids or other approved decontaminants may yield quantifiable decreases [125]. They can be decreased due to organic matter and minimal penetration of skin follicles or uneven surfaces [21]. Efficacy may improve with concentration, but this will have an impact on odor, color, worker safety (chemical hazard), corrosiveness, wastewater quality, and regulatory acceptability.

Freezing minimizes culturable populations but should never be sold as a sterilizing process. The nature of this effect depends on the temperature, duration of freezing, food matrix, strain, and freeze–thaw conditions. Chilling is mainly a multiplication‐restriction agent and has only a modest inactivation ability [126].

The highest commercial validation is for hygienic process control, optimized chilling, thermal treatments, and selected chemical interventions. However, this is a side effect when we talk about the total remaining in each step of the line and recontamination after treatment. If equipment, process water, or subsequent handling reintroduces the organism, a large immediate reduction may have little final benefit.

### 7.6. Natural Antimicrobials, Coatings, Packaging, and Nano‐Enabled Systems

Plant‐derived antimicrobials, such as essential oils, organic acids, bacteriocins, peptides, and phenolic compounds, can inhibit *C. jejuni* activity by disrupting the membrane [127]. Nonetheless, when in contact with complex food matrices, the activity that manifests when tested in isolation can be attenuated due to interactions with proteins or lipids present in the matrix, pH variation, and/or the physical structure of the food [128].

The effectiveness of compounds such as carvacrol, thymol, and eugenol depends on their concentration, which can affect sensory quality [129]. Stability and reduction of sensory effects can be achieved for ENM by encapsulation, edible coatings, and controlled‐release packaging; however, scale‐up, migration, cost, and regulatory issues remain challenging [130, 131]. Nano‐enabled systems appear promising, but major challenges need to be addressed in terms of the evaluation of particle characteristics (such as size, shape, and charge), release behavior from the food matrix, efficacy within the food nematode/living system, safety, and regulatory applicability prior to commercial application.

### 7.7. Consumer and System‐Level Interventions

Although adequate cooking serves as the ultimate effective step for inactivation, consumer‐level protection relies on the prevention of cross‐contamination. The separation of raw and ready‐to‐eat foods, handwashing, cleaning of utensils and surfaces, prevention of package leakage, and avoidance of washing raw poultry can reduce pathogen transfer in domestic kitchens [132].

The effectiveness of consumer education is difficult to express as a bacterial log reduction because outcomes depend on behavior, knowledge retention, kitchen design, and actual contamination levels. Controlled kitchen studies may quantify transfer rates; however, these do not always predict long‐term behavioral change [36]. Messages should be specific, feasible, and grounded in observed high‐risk practices.

System‐level interventions include antimicrobial stewardship, process performance criteria, integrated surveillance, environmental management, and quantitative microbial risk assessment [6]. These approaches rarely generate a direct laboratory reduction value but can influence resistance selection, compliance, outbreak detection, and cumulative population risk. Their effectiveness should be assessed through longitudinal surveillance and disease incidence outcomes.

### 7.8. Comparative Assessment and Multihurdle Integration

Preharvest interventions can reduce *C. jejuni* loads entering slaughter but often show variable or transient efficacy, whereas processing interventions are generally more controllable but may be limited by product quality, regulatory constraints, and recontamination [133]. Consumer protection ultimately depends on adequate cooking and prevention of cross‐contamination [36]. Thus, effective control requires a multihurdle approach integrating validated farm biosecurity measures, biological controls where available, hygienic processing, decontamination strategies, cold‐chain management, and *C. jejuni* surveillance at critical points throughout the farm‐to‐fork continuum. Further research is required to understand whether these intervention effects cascade down the food chain and result in reductions of human disease that are meaningfully large enough to be clinically significant. Table 3 summarizes comparative efficacy, durability, translational maturity, and implementation barriers.

**Table 3:** Preharvest, processing, storage, retail, and domestic interventions for *C. jejuni*, showing primary efficacy outcomes, durability, translational maturity, and major limitations across the food‐production continuum.

Intervention	Stage and model	Primary outcome and representative efficacy	Durability	Translational maturity	Interpretive/implementation limitations	References
Biosecurity and flock management	Preharvest; commercial or experimental flocks	It reduces flock positivity or delays colonization.	This may persist through the flock cycle with consistent implementation.	Established; implementation‐dependent	Adherence, farm design, insects, water, equipment, cleaning, and environmental reintroduction.	[105, 106]
Microbiota‐directed interventions	Preharvest; experimental broilers	Effects ranged from negligible to several‐log cecal reductions under controlled conditions.	The results were often variable or transient.	Moderate; product‐specific	Formulation, strain or consortium, dose, timing, diet, resident microbiota, challenge strain, and stability were also considered.	[108, 109]
Bacteriophages	Preharvest; usually near slaughter	Frequently produces rapid short‐term cecal reductions.	Usually, hours to days; rebound may occur.	Moderate	Host range, dose, timing, gut stability, resistance, field strain diversity, and limited commercial validation.	[116, 117, 121]
Vaccines	Preharvest; experimental broilers	Outcomes ranged from no measurable effect to substantial colonization reduction.	Uncertain: constrained by the short broiler cycle.	Preliminary	Antigenic variation, maternal antibodies, route, adjuvants, challenge strain, and delayed immunity were also considered.	[22, 105, 120]
Chemical decontamination	Processing; carcasses, skin, or process water	It reduces viable surface contamination under controlled conditions.	Immediate recontamination may reduce the benefit.	Moderate; selected treatments established	Organic load, follicle penetration, sensory effects, corrosion, wastewater, regulation, and line compatibility were assessed.	[122, 126]
Thermal or steam treatments	Processing; carcass surfaces	It provides substantial immediate inactivation when optimized.	Immediate if recontamination is prevented.	Moderate	The effects of nonuniform heating, line speed, energy, appearance, texture, moisture, and yield were also investigated.	[123, 124]
Cold‐chain control and freezing	Storage and distribution; poultry products	Chilling restricts bacterial multiplication, whereas freezing partially reduces the number of recoverable cells.	Storage‐dependent.	Established operationally	Does not eliminate contamination; effects vary by strain, matrix, temperature, duration, and freeze‐thaw history.	[21, 32, 33]
Natural antimicrobials and coatings	Processing or storage; meat, skin, or coatings	Activity is usually stronger in broth than in complex food matrices.	Concentration‐ and storage‐dependent effects.	Preliminary	Matrix interference, instability, sensory effects, authorization, and consumer acceptance are also important factors.	[127, 128, 130]
Active packaging and nano‐enabled delivery	Storage and retail; films or controlled‐release systems	This may reduce the survival or recoverable populations during storage.	Formulation‐ and release‐dependent effects.	Emerging	Migration, toxicity, release variability, scale‐up, recyclability, cost, and regulations.	[130, 131]
Consumer thermal and hygiene controls	Domestic preparation	Cooking effectively inactivates cells, and hygiene reduces cross‐transfer.	Immediate for cooking; behavior‐dependent for hygiene purposes.	Established for cooking; moderate for implementation	Time–temperature control, kitchen practices, adherence, and risk communication.	[5, 32, 34, 36]

*Note:* Outcome metrics differ between intervention stage and study design, and thus the efficacy measures are not directly comparable. Quantitative values are reported where comparable evidence exists; otherwise, qualitative outcomes are retained.

## 8. Detection, Genomic Surveillance, and Source Attribution

The accurate detection and genomic surveillance of *C. jejuni* are essential for epidemiological investigation, source attribution, and control. Since different methods provide varying insights into culturability, viability, identity, and strain‐relatedness, it is wise to employ a combination of techniques instead of depending on a single assay.

### 8.1. Culture and Molecular Detection

Culture is important because it validates recoverable viable organisms and allows isolates to be used for antimicrobial‐susceptibility testing, typing, and sequencing. Nonetheless, cell injury, competing microbiota, selective conditions, and the VBNC state may decrease recovery [134]. PCR and qPCR allow for quicker species‐specific detection but are dependent on extraction efficiency, inhibitors, assay specificity, and food or environmental matrix effects [135, 136]. A key limitation is that molecular assays may result in the detection of DNA from nonviable cells. Despite the reduction in amplification from membrane‐compromised populations, viability‐PCR approaches do not achieve full differentiation of viable, injured, VBNC, and dead cells [134, 137]. The strengths and limitations of the detection methods frequently used in practice are shown in Table 4.

**Table 4:** Main limitations, viability assessment, culture requirements, and applications including operation characteristics, analytics outputs, and turnaround times of methods for the detection, identification, and monitoring of *C. jejuni*. information.

Method	Principal output	Typical turnaround	Sensitivity depends mainly on	Viability information	Culture requirement	Main application	Principal limitation	Reference
Selective culture	Recoverable isolate	2–5 days	Matrix, enrichment, medium, incubation, and cell injury	Recoverable culturability	Produces an isolate	Confirmation, enumeration, AST, and sequencing	This method is slow and may miss injured or VBNC cells.	[138]
Conventional PCR	Qualitative target detection	Several hours	Extraction, inhibitors, target, sample volume, and enrichment	None	Not required	Rapid species confirmation	Nonquantitative; may detect dead cell DNA.	[139, 140]
Real‐time PCR	Calibrated quantitative or semiquantitative signal	2–6 h	Extraction, standards, inhibitors, efficiency, and enrichment	None unless viability treatment is added	Not required	Rapid screening and calibrated quantification	Ct values require validated standards and control.	[134, 135]
Multiplex PCR	Simultaneous detection of multiple targets	Several hours	Primer compatibility, target abundance, inhibition, and assay design	None	Not required	Species, virulence, or resistance‐target screening	Primer competition and incomplete matrix validation.	[139]
Viability PCR, including PMA‐qPCR	Signal enriched for membrane‐intact cells	4–8 h	Dye treatment, light exposure, matrix, cell density, and membrane injury	Partial; membrane integrity only	Not required	Assessment of stressed populations	Does not establish infectivity or complete viability.	[134, 137]
Whole‐genome sequencing	Genome, AMR determinants, virulence profile, and relatedness	Several days after isolation	DNA quality, depth, assembly, contamination, and analysis pipeline	Indirect	Usually requires an isolate	Outbreak investigation, source attribution, and AMR surveillance	It requires infrastructure, bioinformatics, quality assurance, and metadata.	[141, 142]

### 8.2. Whole‐Genome Sequencing and Source Attribution

High‐resolution characterization of *C. jejuni* populations and support for outbreak investigation, as well as comparison of isolates from animals, food, environments, and human infections, can be achieved through whole‐genome sequencing. Genomic similarity does not provide evidence of direct transmission but should be interpreted in conjunction with temporal, geographical, epidemiological, and production‐chain metadata [143, 144]. Isolate recovery, sequencing, and bioinformatics capacity, quality assurance, and methodological harmonization continue to limit broader implementation. Figure 4 shows integration of detection, genomic surveillance, and source attribution.

**Figure 4:**
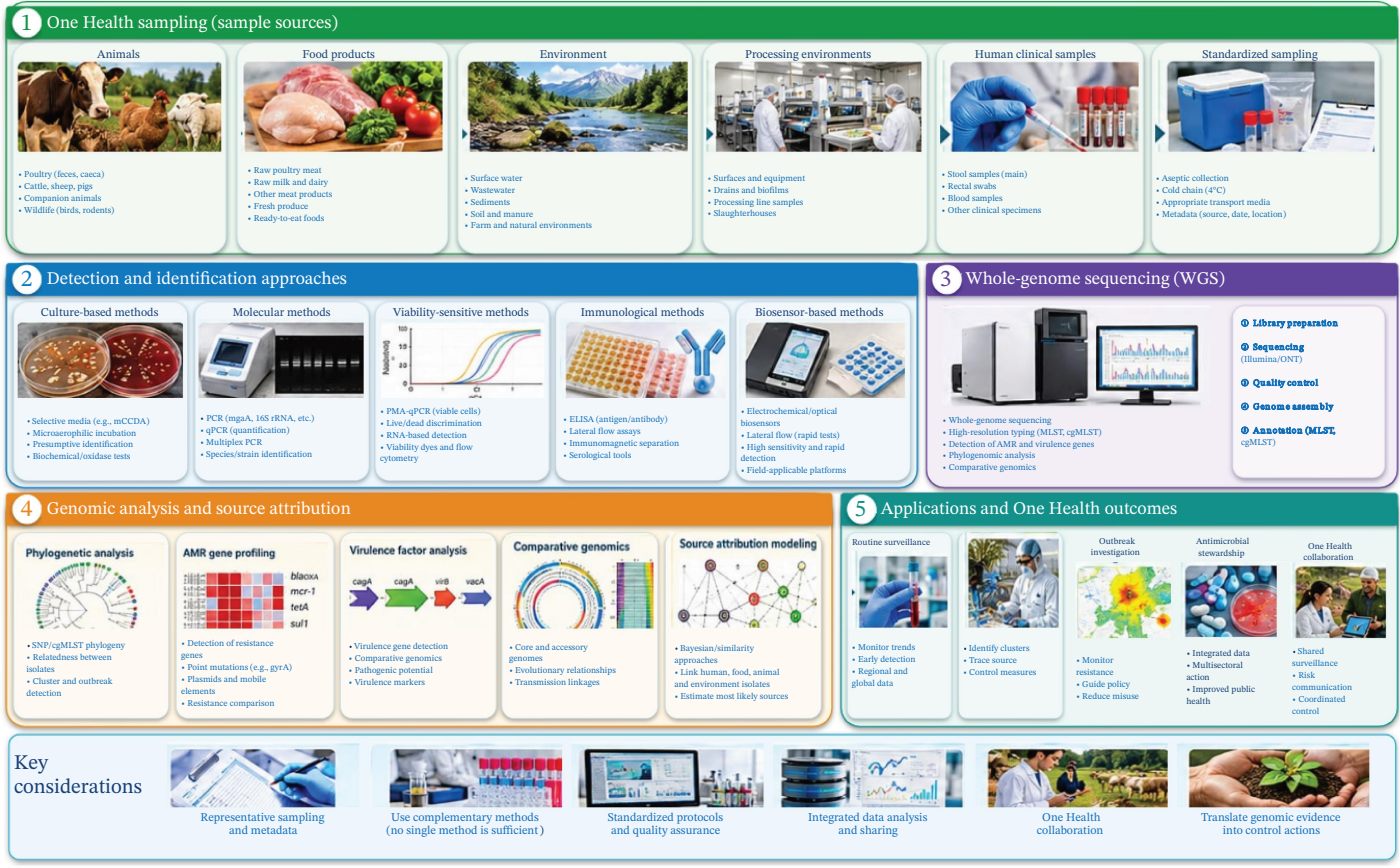
Detection, genomic surveillance, and source attribution of *Campylobacter jejuni.* Schematic overview of sampling through complementary culture‐based, molecular, viability‐sensitive, immunological, and biosensor detection methods using whole‐genome sequencing and genomic analyses for source attribution.

## 9. Integrated Knowledge Gaps and Research Priorities

Future studies should progress from stand‐alone laboratory observations to integrated transmission‐linked evidence across the food‐chain continuum. Longitudinal studies that incorporate farms, processing environments, retail foods, and reservoirs in the environment, especially human cases, are essential to identify where *C. jejuni* amplification or interruption occurs. Geographical diversity, especially in regard to informal markets, smallholder systems, and low‐resource settings, is equally important, where raw milk, untreated water, wildlife, and livestock contact may play a different or even major epidemiological role.

Intervention studies would benefit from more harmonized and comparable endpoints and greater commercial validation. Cecal counts, carcass prevalence, surface contamination, cross‐contamination, product quality, cost, and predicted human risk should not be considered interchangeable endpoints. We therefore recommend that promising phages, probiotics, vaccines, and antimicrobials be tested on multiple strains, farms, seasons, and commercial conditions with both successful and neutral results reported together with their effect sizes.

Prioritizing this research within surveillance and analytical approaches includes improved characterization of VBNC populations and the integration of phenotypic and genomic AMR data. Culture, viability‐sensitive molecular methods, genomic prediction, and standardized susceptibility testing should not be interpreted separately but should be merged. Future preharvest containment measures should also include interventions that are amenable to commercial broiler production and which consider microbiota stability (i.e., microbiota‐directed products), the development of quickly acting protective vaccines, along with phage strategies that account for host range and resistance. Additive or synergistic effects may be assumed to occur in multihurdle combinations, but should be demonstrated experimentally. Injuries to animals need to establish the mechanisms of toxicity.

## 10. Limitations of This Review

This review has several limitations that need to be acknowledged, when drawing conclusions from it. In the first instance, because a narrative approach rather than a systematic review was taken, despite balancing efforts to include evidence across the farm‐to‐fork continuum it may be that not all studies published in the literature are represented. Second, the review mainly relied on English‐language publications, which can also lead to language and publication bias as well as by failing to account for potentially relevant evidence published in other languages. Third, uneven geography of evidence, with substantially more literature on intensive poultry‐production systems and resource‐rich surveillance settings than smallholder production, informal food markets, conflict‐affected areas and resource‐poor settings. As a result, the relative contribution of specific reservoirs, transmission routes, patterns of antimicrobial resistance, and intervention effectiveness may not be directly comparable between regions. Lastly, a lack of standardization between study designs including sampling strategies, detection methods, definitions for outcomes and experimental or commercial conditions restricts the ability to compare findings directly across studies. This emphasizes the importance of studies over a range of geographical locations, harmonized surveillance and reporting as well as a standardized approach to outcome measures along the farm‐to‐fork continuum in *C. jejuni* research.

## 11. Conclusion

*C. jejuni* continues to thrive as a leading foodborne pathogen because of the interplay between efficient colonization of animal reservoirs, cross‐contamination during processing, survival under multiple stressors, virulence potential, and antimicrobial resistance, coupled with repeated opportunities for transmission across the food chain. In many settings, poultry is the major reservoir for human exposure, although ruminants, raw milk, water, wildlife, and environmental exposure pathways can also contribute a large fraction of human exposures. Consequently, control is unlikely to be effective when it relies solely on one measure but requires concerted interventions at multiple points during preharvest, processing, retailing, and consumption. New strategies need to be evaluated based on lab success, but also reproducibility, the ability to commercialize them safely, and whether they can mitigate human risk. Future advances will rely on integrated One Health surveillance, harmonized intervention effects, and research that connects these molecular or microbiological findings with their putative roles in transmission and the impact of disease.

## Author Contributions

**Othman Abdulrahman Mohammed:** conceptualization, methodology, software, investigation, data curation, formal analysis, writing – original draft, and writing – review and editing. **Mohammed Ali Hama:** methodology, investigation, data curation, formal analysis, and writing – original draft. **Saman A. Mohammed:** investigation, software, data curation, formal analysis, and writing – review and editing. **Musa Muatasam Zorab:** methodology, investigation, and writing – review and editing. **Suhaib H. Kakamad:** methodology, investigation, and writing – review and editing. **Hazha Hassan Tahir:** investigation, data curation, and writing – review and editing. **Amirreza Gholami:** methodology, software, formal analysis, and writing – review and editing. **Azad Mohammed Aziz:** investigation, data curation, and writing – review and editing. **Hamza Kamaran Hama:** investigation and writing – review and editing. **Kawther Hoshear Faiq:** investigation, data curation, and writing – review and editing. **Ammar Alfergah:** conceptualization, supervision, project administration, and writing – review and editing.

## Funding

No funding was received for this manuscript.

## Disclosure

All authors read and approved the final manuscript.

## Conflicts of Interest

The authors declare no conflicts of interest.

## Data Availability

Data sharing is not applicable to this article as no datasets were generated or analyzed during the current study.
